# Outcomes in BCG failure: Outcome from a single centre UK experience

**DOI:** 10.1002/bco2.70025

**Published:** 2025-05-06

**Authors:** Elizabeth Day, Rachel Aquilina, Lazaros Tzelves, Ashwin Sridhar, Anthony Ta, John Kelly, Bernadett Szabados

**Affiliations:** ^1^ Department of Urology University College London Hospital NHS Foundation Trust London UK; ^2^ School of Medicine University College London London UK; ^3^ 2nd Department of Urology Sismanogleio Hospital, National and Kapodistrian University of Athens Greece; ^4^ Department of Urology St Vincent's Hospital Melbourne Australia; ^5^ Barts Experimental Cancer Medicine Centre, Barts Cancer Institute Queen Mary University of London London UK

**Keywords:** BCG failure, bladder cancer, bladder sparing treatment, Hyperthermic Mitomycin, radical cystectomy

## Abstract

**Objective:**

To describe real‐world outcomes of patients with BCG failure undergoing bladder‐sparing treatments (BSTs) vs radical cystectomy in the UK.

**Patients and Methods:**

A single institution audit was conducted at a tertiary bladder cancer referral service (UCLH, London, UK). Patients with BCG failure treated between January 2017 and September 2022 were included. BSTs included endoscopic surveillance, hyperthermic mitomycin and further BCG. The primary outcome was event free survival (EFS). Complete response (CR) rate and duration of response (DoR) were investigated in patients undergoing BST. The secondary outcomes were 3‐ and 5‐year cancer‐specific (CSS) and overall survival (OS).

**Results:**

A total of 112 patients were included: 30% (34/112), 32% (36/112) and 27% (30/112) had BCG unresponsive, exposed and intolerant disease and 11% (12/112) had progressed to muscle invasive disease (MIBC).

In the BCG unresponsive and exposed groups, 79% (27/34) and 72% (26/36) underwent RC, with the remaining receiving BSTs. Comparing RC vs BST in BCG unresponsive and exposed groups combined, there was a significantly poorer EFS in the BST group (p < 0.001); 35.3% (6/17) patients transitioned to second‐line BST due to recurrence or intolerance and a further 50% (3/6) transitioned a third line BST. There was no significant difference in CSS or OS rates. In BCG intolerance, the EFS rate was 90% as three patients experienced high‐grade recurrence and underwent RC. There were no cancer‐related deaths. In MIBC group, 5/12 presented with metastatic disease and 3‐ and 5‐year CSS rates was 66% and 0%.

**Conclusion:**

This data reports real‐world practice in a UK centre. BSTs in BCG unresponsive and exposed disease are supported as an alternative to RC providing the increased risk of recurrence is accepted. Additionally, consideration of formal guidance supporting BST is needed in BCG intolerance, which appears to have an excellent outcome in a cohort managed with endoscopic surveillance. Upstaging to MIBC remains a poor prognostic factor and is key to improving survival outcomes in BCG failure.

## 1 INTRODUCTION

Radical cystectomy (RC) is the gold standard treatment for patients with high‐grade bladder cancer recurrence during or after intravesical Bacillus‐Calmette Guerin (BCG) therapy.^1^ However, approximately 50% of patients are either unfit for or decline major surgery and opt for alternative bladder‐sparing treatments (BSTs).^2^ To date, there are three FDA‐approved therapy agents in this setting, however, BST options are more limited in the UK and Europe.^1^ In the UK, options include hyper‐thermic intravesical mitomycin, BCG re‐challenge, endoscopic surveillance and enrolment in clinical trials.

Additionally, there is very limited evidence on the optimal approach in patients who do not tolerate BCG treatment with current European Guidance unable to provide a recommendation for this group^1^

As a number of new agents are currently being investigated in clinical trials there is an expectation that BST options in the UK and Europe will expand. However, there is limited contemporaneous real‐world data to compare the efficacy of these new options to the current practice. The aim of this work is therefore to describe outcomes of patients with BCG failure or intolerance in a UK centre.

## 2 PATIENTS AND METHODS

A retrospective audit of all patients diagnosed with BCG failure between January 2017 and September 2022 was conducted at a tertiary bladder cancer referral service (University College London Hospital NHS Foundation Trust, London, UK). This work was performed within the context of a service improvement project and registered with the local institutional board (UROONC202409).

BCG failure is generally defined as any high‐grade (HG) recurrence during or after BCG therapy. In this study, patients were grouped by the European Association of Urology definition and included the BCG unresponsive group recommended by the FDA and the BCG exposed group that encompasses a further group of high‐risk patients that do not meet the unresponsive criteria.^1^, ^3^ BCG intolerance encompasses those patients who experience severe side effects that prevent continuation of treatment. The muscle‐invasive bladder cancer (MIBC) group included all patients diagnosed with MIBC in the follow‐up period. Further details of the classification used are available in Supplementary Material – Table 1.

### 2.1 Treatment

Treatment options in BCG failure were recommended via a multidisciplinary team meeting and discussed with the patient to come to a shared decision. At the diagnosis of BCG failure, it is standard practice for patients to be recommended radical cystectomy but this may not be pursued due to patient choice or the patient being assessed as unfit for major surgery. Bladder‐sparing options would then be explored.

Available BSTs include device‐assisted intravesical hyperthermic mitomycin (HIVEC®, Combat Medical and Synergo®RITE, Medical Enterprises Group), BCG re‐challenge and endoscopic surveillance alone.

The BCG regimen employed at our institution for both primary management and re‐challenge includes 6× weekly induction, followed by 3× weekly maintenance treatments according to the Lamm protocol for up to 3 years.^4^

The HIVEC and Synergo regimens employ a 6× weekly induction followed by a six‐week break. A single maintenance installation is then given every 6 weeks until treatment is discontinued.

Endoscopic surveillance is carried out according to the EAU guidelines.^1^ Recurrences are treated with endoscopic management, in our centre this includes transurethral resection and laser ablation.

Patients treated with RC received regular cross‐sectional imaging surveillance during the follow‐up period. Patients undergoing BST underwent endoscopic and imaging surveillance according to the EAU guidelines.

### 2.2 Outcomes

The primary outcome was event‐free survival (EFS). In keeping with FDA recommendations for NMIBC,^5^ an event is defined as persistence of CIS at 6 months, development of any HG Ta/CIS/T1 within the bladder or remaining urinary tract in patients undergoing BST, development of muscle‐invasive, advanced bladder cancer or any death due to cancer. For BSTs, complete response (CR) rates at 3 and 6 months, median duration of response in patients with CIS (mDoR) and cystectomy‐free survival rate are also reported in line with recommendations.^5^ Day 0 in BST groups for complete response is three months from starting BST at the time of the first check cystoscopy.

The secondary outcomes were 3‐ and 5‐year cancer‐specific survival (CSS) and overall survival (OS). CSS is defined as the time between diagnosis of BCG failure and cancer‐related death. OS is defined as the time between diagnosis of BCG failure and death from any cause.

Patients without an event were censored at the date of their last follow up.

### 2.3 Statistical analysis

Patient groups were compared using descriptive statistics. Survival was estimated using the Kaplan‐Meier method, and distributions within subgroups were compared using the log‐rank test. P values of <0.05 were considered statistically significant. All statistical analyses were performed in SPSS v29. Due to the small number of bladder‐sparing treatments, unresponsive and exposed groups were combined in the analysis.

## 3 RESULTS

Between January 2017 and September 2022, 112 patients were diagnosed with BCG failure or intolerance. Baseline clinicopathological characteristics are given in Table 1. The median follow‐up for the entire cohort was 39.3 months (95% CI: 35.0–43.6). Outcomes by BCG Failure group are given in Table 2.

**TABLE 1:** Clinico‐pathological characteristics of patients in BCG groups.

	BCG unresponsive (n = 34)	BCG exposed (n = 36)	MIBC (n = 12)	BCG intolerance (n = 30)
**Definition**	Persistent CIS or HG Ta/T1 Initial clearance after adequate BCG but subsequent recurrence within 6mo (for HG Ta/T1) or 12mo (for CIS)	HG Ta or CIS at 3mo after induction BCG Delayed relapse (>24mo)	Progression to MIBC at BCG failure	BCG discontinued due to toxicity
**Median age, yrs**	72	72	68	72
**Male**, n (%)	29 (88%)	28 (78%)	10 (83%)	24 (80%)
**Time to BCG Failure, months** (median, IQR)	9.6 (7.4–13.2)	27.0 (18.5–41.7)	18.0 (11.8–23.2)	45.0 (26.2–61.3) *
**T stage, n (%)**	**At diagnosis of BCG failure**			**At first diagnosis**
**Ta**	7 (20%)	17 (47%)	NA	15 (50%)
**T1**	22 (65%)	8 (24%)	NA	12 (40%)
**CIS alone**	5 (15%)	10 (29%)	NA	3 (10%)
**Concomitant CIS**	23 (96%)	23 (64%)	4 (33%)	15 (50%)
**≥ T2**	NA	NA	12 (100%)	NA
**Metastatic**	NA	NA	5 (42%)	NA

BCG = Bacillus Calmette‐Guerin; CIS = carcinoma in situ; IQR = interquartile range; MIBC = muscle‐invasive bladder cancer.

Table 1. Baseline clinical and pathological characteristics of patients who failed BCG therapy and underwent subsequent radical cystectomy or bladder‐sparing treatment.

* The time from discontinuing BCG due to toxicity to high‐grade cancer recurrence.

**TABLE 2:** Outcomes in BCG failure groups by radical cystectomy and bladder sparing treatments.

Outcomes	BCG unresponsive		BCG exposed		BCG intolerance	MIBC
**Treatments received, n (%)**	**RC** 79% (27/34)	**BST** 21% (7/34)	**RC** 72% (26/36)	**BST** 28% (10/36)	**BST: surveillance** 100% (30/30)	**RC** (n = 4) **Chemotx** (n = 5) **palliative** (n = 3)
**EFS rate**	96% (26/27)	29% (2/7)	89% (23/26)	40% (4/10)	90% (27/30) *	25% (1/4) **
**24‐mo EFS rate**	96% (26/27)	29% (2/7)	92% (24/26)	40% (4/10)	100% (30/30)	50% (2/4)
**CR rate at 3 m in CIS**	NA	43% (3/7)	NA	86% (6/7)	NA	NA
**CR rate at 6 m in CIS**	NA	29% (2/7)	NA	71% (5/7)	NA	NA
**mDoR in CIS (95%CI)**	NA	4.6 (0.0–13.0)	NA	16.2 (2.8–29.7)	NA	NA
**Cystectomy‐free survival rate**	NA	71% (5/7) ***	NA	100% (10/10)	90% (27/30) *	NA
**3‐year CSS**	92% 12/13	83% (5/6)	94% (16/17)	100% (4/4)	100% (17/17)	66% (4/6)
**5‐year CSS**	75% (3/4)	50% (1/2)	71% (5/7)	75% (3/4)	100% (8/8)	0% (0/3)
**3‐year OS**	92% (12/13)	66% (4/6)	75% (15/20)	80% (4/5)	77% (17/22)	33% (3/9)
**5‐year OS**	50% (2/4)	33% (1/3)	42% (5/12)	40% (2/5)	62% (8/13)	0% (0/9)

BCG = Bacillus Calmette‐Guerin; BST = bladder‐sparing therapy; Chemotx = chemotherapy; CIS = carcinoma in situ; CR = complete response; CSS = cancer‐specific survival; mDOR = median duration of response; MIBC/M1 = muscle‐invasive or metastatic bladder cancer; OS = overall survival; RC = radical cystectomy.

Table 2 **‐** EFS rate: Events include persistence of CIS at 6 months, development of any HG Ta/CIS/T1 within the bladder or remaining urinary tract, or development of muscle‐invasive, advanced bladder cancer or death due to cancer. EFS rate in MIBC: recurrence amongst those who have had RC (n = 4). CR = complete response (absence of high‐grade recurrence)DoR: duration of CR: defined as the time from the first response (at 3 months) to disease progression or high‐grade recurrence.

* Three patients with high‐grade recurrences underwent cystectomy.

** only patients who underwent RC. Three patients developed metastatic disease after undergoing RC.

*** two patients underwent radiotherapy for MIBC.

### 3.1 BCG unresponsive and exposed groups combined

**FIGURE 1:**
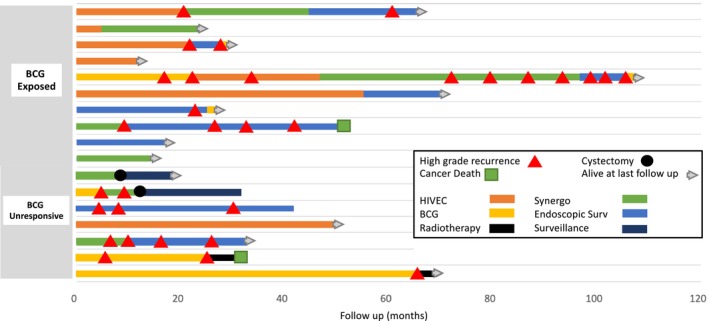
**Bladder sparing treatments in BCG unresponsive and BCG exposed groups.** Swimmer plot describing the outcomes of patients receiving bladder‐sparing treatments in both BCG unresponsive and exposed groups.

A total of 70 patients had BCG unresponsive or exposed disease. Seventy‐six percent (53/70) underwent RC and 24% (17/70) received BSTs. Of the patients receiving BSTs, 64.7% (11/17) patients were deemed unfit for cystectomy and 35.3% (6/17) patients declined cystectomy. The types of BST selected, and subsequent treatments are described in **Figure** **1**; 35.3% (6/17) patients undergoing first‐line BST transitioned to second‐line treatment due to high‐grade NMIBC recurrence or treatment intolerance and a further 50% (3/6) transitioned to a third line BST.

#### 3.1.1 Bladder sparing treatments vs radical cystectomy

**FIGURE 2:**
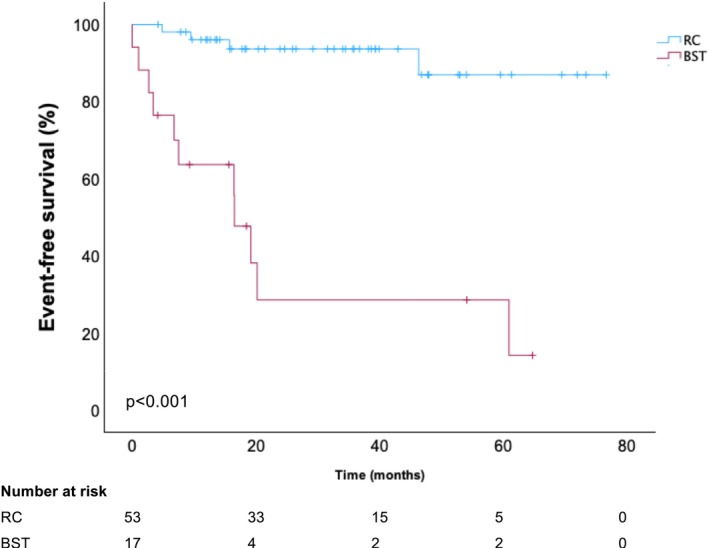
**Event free survival for radical cystectomy vs bladder sparing treatments in in BCG unresponsive and exposed groups combined**. Kaplan–Meier curves for EFS for RC vs BSTs in BCG unresponsive and exposed groups combined.

Event free survival was significantly longer for patients undergoing radical cystectomy vs. bladder‐sparing treatment with 70.4 months (95% CI 64.6–76.3) vs. 25.7 months (95% CI 12.5–39.0, p < 0.001), respectively (Figure 2). In the BST group, 17.6% (3/17) patients progressed to MIBC and 5.9% (1/17) patients to metastatic disease. In the RC group, 7.5% (4/53) of patients progressed to metastatic disease.

There was no significant difference in 3 and 5‐year CSS and OS between for the bladder‐sparing treatment vs radical cystectomy groups (Table 2, CSS p < 0.73 and 0.91, OS p < 0.51 and 0.77).

#### 3.1.2 Outcomes in patients receiving bladder sparing treatments

Complete Response is calculated for patients with CIS; 83% (14/17) of patients treated with BSTs had CIS at the time of BCG failure. CR rate was 64% (9/14) at 3 months and 50.0% (7/14) at 6 months. The median duration of response of patients treated with BSTs in the BCG unresponsive and BCG exposed groups were 4.6 months (95% CI 0.0–13.0) and 16.2 months (95% CI 2.8–29.7) months, respectively.

The cystectomy‐free survival rate overall for all patients receiving first‐line BST was 88.2% (15/17). In BCG unresponsive patients, is was 71% (5/7) and 100% (10/10) in BCG exposed. However, in the BCG unresponsive group, two patients underwent radiotherapy for MIBC.

### 3.2 BCG intolerance

A total of 27% (30/112) developed BCG intolerance. 77% (23/30) and 23% (7/30) of patients with BCG intolerance received adequate and inadequate BCG therapy, respectively. Of the seven patients with inadequate BCG, the number of doses ranged from 1 to 6. The predominant symptoms leading to BCG cessation were lower urinary tract symptoms (56%) and arthritis (23%).

The overall EFS rate was 90% as three patients experienced high‐grade recurrence and underwent RC. All recurrences occurred more than 24 months after BCG was discontinued (Table 2). There were no low‐grade recurrences in this group. The 3‐ and 5‐year CSS rates were 100% (Table 2).

### 3.3 Muscle invasive disease at the time of BCG failure

A total of 11% (12/112) of patients had disease progression at the time of BCG failure. Of these 6% (7/112) had muscle‐invasive disease (MIBC) and 57% (4/7) of them underwent RC. One patient received radiotherapy and the remaining two patients received the best supportive care due to their fitness and comorbidities. From the four patients who underwent RC, 75% (3/4) progressed to metastatic disease (Table 2). At the time of BCG failure, 5% (5/112) of patients had progressed further to metastatic disease and received systemic therapy. The 3‐ and 5‐year CSS rates was 66% and 0%.

## 4 DISCUSSION

Our report describes the outcome of patients with BCG unresponsive or intolerant urothelial carcinoma of the bladder. To our knowledge, this is the largest cohort describing real‐world outcomes in this patient population in the UK.

In BCG‐exposed and unresponsive disease, the current European and UK guidelines recommend patients with BCG failure undergo radical cystectomy.^1^ However, some patients are either unfit or refuse to undergo surgery. In these patients, alternative options with bladder‐sparing therapies are utilized. In our patient cohort, the majority (76%) of patients underwent surgery, as per standard of care. The remaining cohort received BSTs which included hyperthermic mitomycin, BCG re‐challenge and endoscopic surveillance. The key difference seen between the BST and RC groups is event‐free survival. Patients receiving BSTs were more likely to experience disease recurrence and this resulted in changes to their BST approach. There was no statistically significant difference in cancer‐specific survival between patients in the groups. This supports observations reported by other groups that bladder‐sparing strategies may be safely employed in a selected patient population and is further underpinned by the recent FDA approvals of pembrolizumab, nadofaragene firadenovec and N‐803.^2^, ^6^, ^7^, ^8^ However, it is likely that the higher rates of events in the BST group impact the patient's overall health‐related quality of life and this should be considered when counselling patients deciding between RC and BSTs. Therefore, our data supports currently available BSTs as a valid alternative to RC when the patient is accepting the increased risk of recurrence.

Regarding the risk of recurrence, the median follow‐up of trials reporting on BSTs, with the exception of Nadofaragene Firadenovec, is shorter than follow‐up in this cohort.^9^ Therefore, the duration of follow‐up of our BST cohort provides valuable insight into the patient journey and can inform the counselling of patients; Over half of patients undergoing first‐line BST transitioned to second‐line BST due to recurrence or intolerance and a further 10% had third line BST. Our practice is to respond to recurrence/intolerance and make use of all BST options available. However, to date, trials have not reported on the use of subsequent BSTs in patients recurring nor is there guidance on the sequencing of BST in the context of recurrence or intolerance of therapy. This is an area that would benefit from further work to inform consensus on the sequencing of BST and indications to switch to an alternative therapy.

Regarding patients presenting with MIBC at the time of BCG failure, in keeping with previously published outcomes these patients had the poorest survival outcomes.^10^ Only a third of MIBC patients in this cohort were able to undergo radical cystectomy with the remainder presenting with metastatic disease or were unfit for surgery. This highlights an area in need of improvement to improve overall bladder cancer survival and should be monitored as novel agents are used first‐line BCG naïve patients.

Finally, regarding the BCG intolerance group, there were no cancer‐related deaths, with 93% avoiding cystectomy. All patients in the BCG intolerance group opted for endoscopic surveillance initially and the outcomes in this cohort suggest that this is a very reasonable recommendation. However, there are currently no guideline recommendations supporting this and the absence of a recommendation is likely due to the paucity of data on this group. The lack of evidence is concerning as BCG intolerance represented 26% of our BCG failure cohort. These patients are therefore being underserved in the research setting and would benefit from further studies to define principles to risk stratify to RC vs BST. In our cohort, over three‐quarters of patients had received adequate BCG and this may therefore be an important prognostic factor. However, due to the absence of events in this group, we cannot draw any conclusions.

This study has a number of limitations. Firstly, its retrospective nature and, secondly, the study period encompasses the covid pandemic which meant that treatment and follow‐up schedules may have been altered when movement restrictions were in place. Thirdly, small numbers in the BST group limited the analysis. The BCG unresponsive and exposed groups were combined to facilitate a more meaningful analysis but we acknowledge that these groups behave differently and most trials to date have focused on the BCG unresponsive group, which limits the ability to compare our real‐world findings to clinical trial data. Additionally, it is challenging to conclude if the study was adequately powered. We performed a posthoc power analysis using the observed hazard ratio and event rates, however with such sparse events in the BST group, this analysis yielded artificially high power estimates (close to 1). This is driven by the inflated hazard ratio from near‐complete separation and does not indicate a genuinely adequate sample size but rather a mathematical artefact of a few events in one arm. Larger, collaborative, studies are therefore required to confirm the observations reported in this study.

## 5 CONCLUSIONS

This data provides real‐world outcomes in BCG Failure from a UK centre using currently available BSTs. The data supports the conclusion that BSTs are a valid alternative to RC in BCG‐exposed and unresponsive patients but has highlighted the need for a better understanding of optimal sequencing of BSTs in the context of recurrence on therapy. Additionally, there is currently a lack of guideline recommendations to support the role of BST in BCG intolerance as this group had excellent outcomes on endoscopic surveillance in this study. Finally, the prevention of MIBC in patients undergoing BCG remains a key issue to improve survival and should be monitored as novel agents are used upfront in the place of BCG.

## AUTHOR CONTRIBUTIONS

*Conceptualization*: Bernadett Szabados. *Data curation*: Rachel Aquilina, Elizabeth Day, Bernadett Szabados. *Formal Analysis*: Elizabeth Day, Bernadett Szabados. *Funding acquisition*: n/a. *Investigation*: Elizabeth Day, Rachel Aquilina, Bernadett Szabados. *Methodology*: Elizabeth Day, Bernadett Szabados, Lazaros Tzelves. *Project administration*: Elizabeth Day. *Resources*: N/A. *Software*: N/A. *Supervision*: Bernadett Szabados. *Validation*: N/A. *Visualization*: Elizabeth Day, Bernadett Szabados, Lazaros Tzelves. *Writing – original draft*: Elizabeth Day. *Writing – review and editing*: Ashwin Sridhar, Anthony Ta, John Kelly, Bernadett Szabados, Lazaros Tzelves.

## CONFLICT OF INTEREST STATEMENT

Nil from any author for the purposes of this work.

## Supporting information


**Data S1.** Supporting Information.
