# Supplementary material for: Outcomes in BCG failure: Outcome from a single centre UK experience
Source: BJUI Compass. 2025 May 6;6(5):e70025. doi: 10.1002/bco2.70025 (PMC12056233; doi:10.1002/bco2.70025)
Supplement: Supplementary file 1 — Data S1. Supporting Information. [file BCO2-6-e70025-s001.docx]

| **BCG unresponsive** |
| --- |
| - T1 HG/G3 tumour present at 3 months. |
| - Ta HG/G3 tumour present after 3 months and/or at 6 months, after either re-induction or first course of maintenance (adequate BCG) |
| - CIS (without concomitant papillary tumour) is present at 3 months and persists at 6 months after either re-induction or first course of maintenance. If patients with CIS present at 3 months, an additional BCG course can achieve a complete response in > 50% of cases |
| - HG tumour appears during BCG maintenance therapy. |
| - T1/Ta HG recurrence within 6 months of completion of adequate BCG exposure or develop CIS within 12 months of completion of adequate BCG exposure |
| **BCG exposed** |
| - BCG-resistant: persistent or recurrent Ta HG and/or CIS disease at three months following at least five of six doses of induction BCG. (These patients have received inadequate BCG so do not fit in unresponsive group.) |
| - Delayed relapse after adequate or inadequate BCG: high-grade recurrence outside of the BCG-unresponsive window and up to 24 months from induction treatment. |
| **BCG intolerant** |
| - Severe side effects that prevent continuation with treatment |
| **Muscle-invasive Bladder Cancer** |
| - Any muscle invasive bladder cancer detected during follow up. |
